# Thrombosis associated with ventriculoatrial shunts

**DOI:** 10.1007/s10143-021-01656-5

**Published:** 2021-10-13

**Authors:** Dengjun Wu, Zhengyan Guan, Limin Xiao, Donghai Li

**Affiliations:** 1 Department of Neurosurgery, The First Affiliated Hospital of Nanchang University, Nanchang, 330006 China; 2 Department of Cardiology, Xiangya Hospital, Central South University, Changsha, 410008 China; 3 Department of Neurosurgery, Jiangxi Provincial Cancer Hospital, No.519 Beijing East Road, Qingshanhu District, Nanchang City, Jiangxi Province 330006 People’s Republic of China

**Keywords:** Thrombosis, Hydrocephalus, Pulmonary embolism, Pulmonary hypertension, Ventriculoatrial shunt

## Abstract

Ventriculoatrial shunts are the most common second-line procedure for cases in which ventriculoperitoneal shunts are unsuitable. Shunting-associated thrombosis is a potentially life-threatening complication after ventriculoatrial shunt insertion. The overall prevalence of this complication is still controversial because of substantial differences in the numbers found in studies using clinical data and in those analyzing postmortem findings. The etiology of thrombosis may be multifactorial, including shunt catheter itself, contents of cerebrospinal fluid, shunt infection, and genetic disorder. The clinical presentation can vary widely, ranging from asymptomatic to a life-threatening condition. Timely recognition of thromboembolic lesions is critical for treatment. However, early diagnosis and management is still challenging because of a relatively long asymptomatic latency and lack of clear guideline recommendations. The purpose of this review is to provide an overview of ventriculoatrial shunt thrombosis, especially to focus on its etiopathogenesis, diagnosis, treatment, and prevention.

## Introduction

Ventriculoperitoneal shunts (VPS) are the conventional first-line choice for treatment of the hydrocephalus, but some circumstances contraindicated for a VPS can preclude the use of the peritoneum, and an alternative distal site is necessary [1]. The most common alternative is the ventriculoatrial shunt (VAS) to the right atrium [2]. However, various complications associated with a VAS have gradually been reported in the literature, more specific of them are cardiopulmonary complications which are potentially life-threatening, including atrial or venous thrombosis with associated pulmonary embolism (PE), pulmonary hypertension (PH), and cor pulmonale [2–8]. What’s worse, only a small proportion of patients with thromboembolism have the perceptible features associated with short-term clinical deterioration while most of VAS recipients may have a long asymptomatic latency after shunt insertion, which may result in misdiagnosis or delayed diagnosis [3, 4, 9, 10]. Therefore, timely identification of such patients and subsequent consideration of intervention are important. Although various treatment options such as surgery, anticoagulation, and thrombolysis have been introduced in the management for patients with VAS-related thromboembolic events, consensus criteria concerning diagnosis, prophylaxis, or treatment are still lacking [9, 11–14]. In this review, we summarize the existing literature regarding the history, epidemiology, etiology, clinical presentation, diagnosis, management, and prophylaxis of VAS-associated thrombosis. Literature has been collected by a search of PubMed using the terms ventriculoatrial shunts, hydrocephalus, thrombosis, thrombus, thromboembolism, pulmonary embolism, pulmonary hypertension, cor pulmonale, and cerebrospinal fluid.

## History of VAS

Gartner in 1896 suggested that the most physiological way for treating hydrocephalus would involve establishing a connection between the ventricles and the venous or lymphatic systems of the head and neck [8]. VAS became the standard treatment for hydrocephalus since 1952. Three years later, in 1955, Scott proposed the first clinically successful iteration of the VPS and introduced the VAS technique [15]. During the early experience, the initial VPS tubing, made of polyethylene, was plagued by unacceptably high rates of peritonitis and distal failure. By comparison, the VAS was the more attractive option at the time, and most cerebrospinal fluid (CSF) shunts of the period were of this design. However, it was quickly noted that VAS were not without drawbacks over the subsequent years. The favorable intervention lead to notable concerns with the recognition of various range of severe and even life-threatening complications due to the specificity of cardiac placement and systemic drainage [1, 8, 16]. Although all ventricular shunts are susceptible to malfunction due to obstruction, disconnection, and infection, patients with VAS are at risk for different complications than those with other types of shunts, the most unique of which are cardiopulmonary complications, such as atrial thrombi, PE, PH, cor pulmonale [2, 4–6, 8]. Conversely, the complications associated with VPS are potentially less morbid and are more easily manageable. By the late 1960s, with the advent of silicone tubing, attention again shifted back to the peritoneal cavity as the preferred terminus for CSF reabsorption. Silicone shunt tubing was shown to have a significantly lower rate of distal obstruction and a less frequent peritonitis compared with the original polyethylene catheters. For these reasons, VAS is increasingly being superceded by VPS as the preferred means of CSF diversion by the 1970s [1]. Even so, when VPS are contraindicated or not successful, there is a notable patient population that remains where VAS is needed [8]. In recent decades, advances in surgical technique, radiographic guidances, and shunt design are expected to reduce the risk of shunt-related complications and to improve the prognosis of patients with hydrocephalus, popularizing the VAS method worldwide.

## Epidemiology

Anderson in 1959 first reported thromboembolism as a complication of VAS [7]. Subsequently there have been many descriptions of this complication in hydrocephalic patients who have undergone shunting procedures, including atrial or venous thrombosis (superior vena cava (SVC), brachiocephalic, subclavian, jugular, and hepatic vein) and PE [12, 14, 17]. However, to date, there is no prospective study on incidence of thrombosis associated with VAS so that knowledge about the actual incidence is limited. The present available documents roughly show that thromboembolic complications present clinically in 0.3% of patients, whereas autopsy series reveal an incidence of up to 60%, and may varies with a time-dependent frequency [10, 12]. Thrombus formation could occur at any time, even decades after VAS insertion [18, 19]. Hemmer found intraoperatively that thrombosis occurred in 20% of patients after indwelling catheters were left in situ for 2 years, in 67% if the catheter was left in for up to 6 years, and in 85% if it was left for up to 14 years [20]. This complication, unique to VAS, could be divided into two principal groups: cardiac and pulmonary thrombi [14]. A right atrial thrombus is the most common cardiac complication. The prevalence of intracavitary thrombi, located in the superior or inferior vena cava, right atrium, and right ventricle, was 34% [10]. PE is another severe complication of the VAS [17]. The incidence of clinically significant PE is 3.2% in patients with VAS. However, these are encountered in 50–100% of patients with VAS at postmortem examination [10, 12, 14]. It means the total incidence of PE during life may be higher, with most emboli remaining clinically undetected, which may lead to potentially increased incidence of PH and cor pulmonale. In VAS patients, PH were recognized clinically in 0.3%, whereas postmortem diagnoses of PH were established in 6.3% [10, 21].

## Etiopathogenesis

**Fig. 1:**
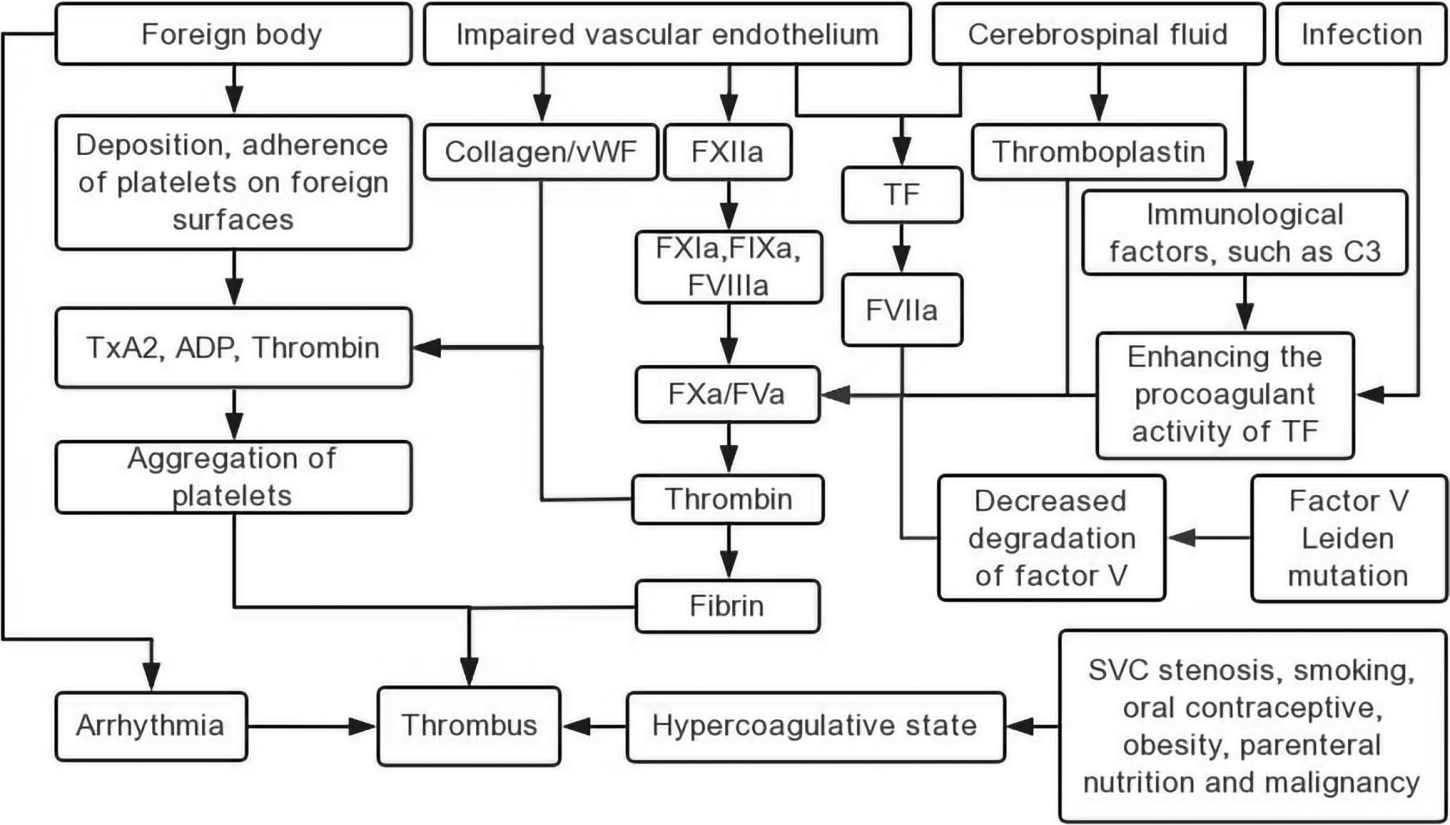
Etiopathogenesis of ventriculoatrial shunt thrombosis. vWF, von Willebrand factor; TxA2, thromboxane A2; TF, tissue factor; SVC, superior vena cava

The etiopathogenesis of VAS-associated thrombosis still remains unclear. Several hypotheses have been put forth to explain the development of thromboembolic events (Fig. 1).

### Foreign body

Extraneous material has a thrombogenic characteristic in case of direct contact to the blood system, and the material properties of the catheter may have a significant influence on thrombosis. Platelet deposition often occurs on all foreign body surfaces which were introduced into the vascular system, and more aggregation takes place in some materials than in others [22]. The VAS catheter may behave as an intravascular foreign body inducing deposition, adherence, and aggregation of platelets and then activation of coagulation cascade, resulting in a fibrinous coating formation on the surface of catheter with subsequent formation of a thrombus [22]. Another possibility is that the catheter damages the vascular endothelium and alters the laminar flow of the blood in the atria or SVC during implantation. Besides, the catheter also could produce a small trauma on the cardiac wall during heart beating, inducing the formation of a thrombus [9, 14].

### CSF

The rate of thromboembolic events in VAS cannot be fully explained on the basis of a foreign body in the right atrium, as patients with pacemaker leads do not have equivalent rates of thromboembolism [23]. It has been suggested that chronic PE leading to PH and death is not due simply to the production of showers of thrombo-emboli, but probably to a reaction of the pulmonary endothelium to some contents of CSF, leading to in situ thrombosis [5, 10, 24]. Some in vitro studies showed that adding CSF to blood makes blood hypercoagulable. They found the accelerated conversion from fibrinogen to fibrin, gradual shortening of the activated clotting time, increased the clot rate, and higher platelet activation after the addition of CSF [25, 26]. Indeed, CSF contains tissue factor and coagulation proteins. Under normal conditions, there is an imbalance between the concentrations of tissue factor and tissue factor pathway inhibitor in CSF, making CSF a procoagulant substance [17]. Besides, coagulation proteins such as thromboplastin and serotonin contained in CSF have been suggested as possible causes of the increased risk of thrombosis [3, 17]. During intracranial hypertension, the release of cerebral thromboplastin in the cerebral ventricle and transport in the VAS could contribute to activation of the coagulation cascade and subsequent thrombus formation [9, 14]. Another important component is serotonin, a potent vasoconstrictor with a controlling role in cell mitosis by the interactions between serotonin and its transporter. These effects may result in proliferation of smooth muscle cell and fibroblast, which plays a critical role in the pathogenesis of severe PH in patients with VAS [4]. Apart from these clotting factors, immunoglobulins and other immunological factors in CSF can cause neuroinflammation, leading to a procoagulant state [17]. In pathological conditions, the concentration of coagulation proteins and the imbalance between tissue factor and tissue factor pathway inhibitor even increase, resulting in a more pronounced procoagulant effect of CSF [26, 27]. However, adding CSF to blood enhances coagulability requiring a starting concentration of 5–9%. Although the concentrations are generally below this critical threshold when CSF is shunted to the venous system, in some specific situations, the concentrated CSF could accelerate clot formation and shunt obstruction [26].

### Infection

Although history of previous shunt infections is found in most patients with thrombi, it remains unclear what role shunt infection plays in the development of a thrombus after VAS implantation [17, 28]. Infection of VAS may be a very important predisposing factor of thrombus formation, as this phenomenon has also been observed in other kinds of intravenous catheters [9]. Bacteria are known to attract complement and clotting factors [14]. Therefore, the presence of latent infection at the site of the shunts could induce a persistent activation of thrombotic factors and coagulation cascade, leading to thromboembolic events [21, 29, 30]. Hyperinflammation with cytokines storm could also be potential mechanism of shunting infection-related hypercoagulable state [31].

### Genetic disorder

Tonn reported a hydrocephalic patient who had undergone VAS procedure developed a large thrombus in the right atrium and subsequently proved to be a positive homozygous factor V Leiden mutation [32]. Coincidentally, in 13 cases with intracardiac thrombosis, all 3 VAS patients who were screened for the factor V Leiden mutation were also found to have a heterozygous mutation [33]. Wilkinson reported that 4 of 5 patients with thrombi were found to have the C677T MTHFR mutations (C to T substitution at position 677) after a period of catheter implantation, which leads to a reduction in enzyme activities involving homocysteine metabolism, also known as hyperhomocysteinemia. The latter is a thrombophilic condition [14]. These suggest that patients with a VAS insertion have an increased risk of thrombophilia as a consequence of genetic disorder.

### Arrhythmia

In VAS patients, the atrial catheter could be abnormally positioned and acted as foci of arrhythmia by irritating the chamber wall [16]. Moreover, high central venous pressures caused by additional volume to the right atrium from the VAS can lead to greater atrial distention and therefore a higher incidence of arrhythmia [34], like atrial fibrillation. The onset of arrhythmia in turn can increase central venous pressure, potentially preventing CSF flow down into the right atrium and cause shunt malfunction and even reflowing of blood [34, 35]. At this juncture, blood will become stagnant, and the risk of intraluminal clot formation is increased [36]. In addition, patients with atrial fibrillation predispose to thrombus formation in the atria and the atrial appendage through a complex interaction among local, systemic, and hemodynamic factors, significantly increasing the risk of systemic thromboembolic events [37].

### Other

The appropriate position of a shunt catheter plays an important role for reducing the incidence of thrombosis. Optimal placement is in the right atrium [1]. When the tip of the catheter moves out of the atrium and into the great vessels, increasing the risk of thrombosis [38]. Other thromboembolic risk factors include SVC stenosis, current smoking, oral contraceptive, obesity, parenteral nutrition, and malignancy [21, 39–42]. Distal tip thrombsis may occur more commonly in patients with these factors because the blood is in hypercoagulative state.

## Clinical presentation

There is a wide variation in the clinical presentation of patients with shunting-related thrombosis, ranging from asymptomatic to a life-threatening condition. Patients primarily complain of shunt malfunction, followed by SVC syndrome, and cardiopulmonary symptoms.

### Shunt malfunction

The most common clinical feature is subacute or chronic-onset shunt malfunction caused by a distal catheter thrombus [43]. Some pathological situations can increase right atrial pressure, frequently as a consequence of PH, and lead to the recurrence of intracranial hypertension characterized by headache, nausea, vomiting, etc. [21]. Patients with normal pressure hydrocephalus may present with the “classic” triad of symptoms of hydrocephalus including psychomotor retardation, gait unsteadiness, and urinary incontinence associated with ventricular dilation [44].

### SVC syndrome

SVC syndrome is a well-known complication to VAS. The basis for this is acute or subacute occlusion of the SVC caused by shunting-related thrombosis [45]. Symptoms of this syndrome vary mainly according to the speed of onset of the obstruction. When obstruction develops slowly and progressively, a collateral circulation develops and symptoms are mild or absent. However, rapid obstruction can lead to significant edema and color changes on the upper extremity, neck, and face, with or without dysphagia, pain, and dyspnea [14, 39]. If the thrombus is located in jugular vein and large enough, it may appear as a neck mass lesion, which may be the only clue of thrombosis [17, 42].

### Cardiopulmonary symptoms

The most lethal complication of VAS is PE and subsequent PH. Detecting a patient with progressing PH is difficult because early clinical findings may be subtle and often be neglected. When the condition is advanced, the patient usually presents with a history of fatigue, coughing (sometimes haemoptysis), chest pain, dyspnea, and febrile episodes [3, 10, 14, 32]. Once symptomatic, PH tends to be relentlessly progressive, developing cor pulmonale and leading to death [5]. Therefore, it has been suggested that regular monitoring for symptoms and signs of cardiopulmonary disease is mandatory, including shortness of breath, dyspnea on exertion, failure to thrive, jugular venous distension, cyanosis, precordial heaves, cardiomegaly, and peripheral edema. Physical examination may show a raised jugular venous pressure with enlargement of the liver and pitting edema of ankles and feet. Auscultation of the heart may reveal a diastolic murmur due to pulmonary valve insufficiency and a systolic murmur due to tricuspid insufficiency [5, 7, 10, 12, 46]. Lack of awareness of an association between progressive respiratory symptom in a VAS and PH may result in delayed diagnosis [4].

### Asymptomatic

The thromboembolic lesions may be relatively asymptomatic, and some patients, even those with severe PH, may remain asymptomatic for prolonged periods [4]. The latency period of cardiopulmonary complications ranges between 10 and 20 years after a VAS insertion [4, 19]. In this period, many emboli lodge in small vessels, giving rise to no symptoms, and some undergo subsequent lysis or recanalization [43]. Therefore, significant embolism may occur with minimal symptoms or signs, and a more common problem is that of repeated “silent” emboli, leading to PH and cor pulmonale [7].

## Diagnose

When the above clinical features present in a patient with a VAS, must raise the suspicion that there could be a thrombus at the distal end of the catheter. However, early clinical diagnosis of thromboembolic complications in patients after VAS insertion could be challenging as signs and symptoms are often subtle and hardly detected by history or physical examination. Especially the prevalence of PE is usually underestimated. Indeed, subclinical or asymptomatic thrombosis can only be diagnosed with advanced imaging. Hence, the diagnosis of VAS-associated thrombosis is typically based on clinical suspicion and imaging confirmation. In those patients with a suspicion of thrombosis, either symptomatic or clinically silent, a multimodality imaging approach may be recommended. In addition to visualization of thrombus, concurrent abnormalities including PE, PH, and cardiac abnormalities should also be assessed.

Echocardiography, especially transesophageal echocardiography (TEE), plays a central role for diagnostic, therapeutic, and prognostic purposes in all patients who underwent VAS. The method can visualize the intracardiac end of the VAS catheter and check its localization and rule out the presence of the thrombus [9]. If a intracardiac thrombus detected, echocardiography can further evaluate thrombus size, mobility, and localization, and provide direct imaging of the wall of thrombus or the appendage of the atrium, which is particularly important in decision-making on optimal treatment strategies [47]. Echocardiographic findings suggestive of PH include right ventricle dilation, interventricular bowing and dyskinesis, plethoric inferior vena cava, tricuspid regurgitation, McConnell sign, and reduced tricuspid annular plane systolic excursion [48]. In patients with confirmed PH, echocardiography can still be performed to estimate the right ventricular systolic pressure or pulmonary artery pressure with continuous wave Doppler, which provides important prognostic information [49]. However, the application of echocardiography in PE and venous thrombosis may be limited so that it is necessary to integrate other images. Computed tomography (CT) with contrast can identify the site, amount of obstruction, and extent of venous blockage [50]. Spiral CT pulmonary angiography, replacing invasive pulmonary angiography as the gold standard exam to diagnose PE, will show multiple perfusion defects caused by chronic micro-embolism and specific diagnostic signs for chronic thromboembolic pulmonary hypertension (CTEPH), such as ring-like stenoses, webs/slits, and chronic total occlusions [3, 48, 49]. Initial testing with chest radiography and pulmonary function test are helpful in excluding parenchymal or airway disease. In PH patients, chest radiography can indicate cardiomegaly, large central pulmonary arteries with rapid distal tapering, and reduced perfusion [40], and an electrocardiogram may demonstrate features of right ventricular and atrial hypertrophy (high voltages, right-axis deviation, and P-pulmonale) and other concurrent cardiac conditions [5, 10, 40].

## Therapeutic strategies

There are currently no standard recommendations for treatment of patients with thrombosis after a VAS placement. Previously reported treatment options including anticoagulation, thrombolysis, and surgery are based on data from a limited number of publications, but the safe and effectiveness of those methods in the resolution of VAS-associated thrombus are still controversial.

### Treatment of atrial or venous thrombosis

VAS is known to be complicated by thrombus formation in the right atrium and SVC system. Those thrombus cannot resolve spontaneously and require removal by intervention as they may lead to PE. There have been occasional case reports of VAS patients with atrial thrombus receiving management of anticoagulation therapy, and the prognosis was reported to be favorable [14, 51, 52]. In patients with SVC thrombosis, anticoagulation is commonly used as primary prevention, especially in patients with severe occlusions [53]. When imaging of the neck, subclavian, and brachial veins identify the presence of thrombus and without risk factors for bleeding, empiric anticoagulation should be advocated [54]. Also of interest, anticoagulation therapy may help to rectify a shunt malfunction and reduce the incidence of distal catheter complications by preventing further clot formation [14, 18, 52].

An increasing body of evidences suggests that intrareservoir administration of thrombolytic therapy can be a useful nonoperative treatment strategy for shunt malfunction associated with thrombsis [11, 55]. Recombinant tissue-type plasminogen activator, streptokinase, and urokinase have been successfully used in management of many patients having an intracardiac thrombus [11, 13, 56, 57]. Timely diagnosis by echocardiography before a thrombus gets bigger or organized leads to effective, safe, and rapid thrombolysis by use of thrombolytic drugs [55]. Notably, intrareservoir administration of thrombolytic drugs may be less efficacious in cases of total distal obstruction or in cases of incompetency of the 1-way mechanism of the shunt valve [11]. There have also been reports of therapeutic failures with thrombolytic therapy, and the mortality rate associated with a medical treatment is high [9], suggesting that a thrombolytic treatment alone may be insufficient because the thrombus is on a foreign body and that it involves the risk of thrombus fragmentation with secondary embolization, especially in cases of large, mobile thrombi [9, 58]. Moreover, the risks of bleeding must be weighed against the benefits. All patients receiving thrombolytics must be followed closely with scans to determine efficacy and clot resolution [55]. In recent years, direct catheter thrombolysis followed by endovascular angioplasty and stenting are emerging modalities of treatment in cases of thrombosis and SVC stenosis secondary to intravenous catheters. These options appear to be very promising and safe alternatives to open surgical bypass [39].

Surgical removal of the thrombus has been the treatment of choice since shunting-related thrombosis was reported. The most direct form of therapy, namely, cardiotomy and removal of thrombus, would presumably be the best treatment [59]. It consisted of a median sternotomy under circulatory arrest, extracorporeal circulation, auriculostomy, embolectomy, and valvular reparation as needed [9, 12]. Atrial and SVC thrombus can be treated successfully by anticoagulants or thrombolysis, but in some circumstances, such as very large and free-floating thrombi, or having an impaired cardiac state, the treatment has to be surgery [60]. In addition, the simple withdrawal of the distal end of a VAS on which there is a thrombus is contraindicated because it would free the thrombus and the risk of embolization would be very high with the possibility of sudden death. Therefore, surgical treatment for these intracardiac thrombi on the distal end of a VAS may be necessary [9]. However, the operative management is complicated, and the surgical mortality associated with this aggressive treatment varies between 21 and 67% [9, 55]. Especially, the risk involved in the presence of chronic cor pulmonale may be prohibitive because the mortality will be 100% [9, 59]. Recently, Dudiy et al. reported a novel technique for percutaneous removal of the right heart thrombi using a suction cannula [61], which has been proven to be is safe, feasible, effective, and avoiding surgical trauma, embolization, and persistent infection [55].

### Treatment of PE, PH, and cor pulmonale

Anticoagulation therapy for confirmed acute PE is the mainstay of treatment. Intravenous administration with low molecular weight heparin, fondaparinux, or intravenous unfractionated heparin is typically used for initial management of inpatients with PE. Discharged patients or those patients suitable for outpatient treatment may be treated with oral anticoagulant drugs [8, 62]. Thrombolytic therapy, including systemic or catheter directed thrombolysis, can be used to accelerate the resolution of acute PE, lower pulmonary artery pressure, and increase arterial oxygenation when a systolic blood pressure persistently less than 90 mm Hg. Bleeding is the major limitation of thrombolytic therapy [62]. Surgical embolectomy with cardiopulmonary bypass can be performed in patients with acute PE associated with hemodynamic instability and contraindication to thrombolytic therapy, but also shows higher mortality [62].

PH and subsequent cor pulmonale due to chronic PE are highly lethal complications of VAS [63]. It has been estimated that, when there is PH, at least 60% of the pulmonary vascularization is already obstructed [9]. Medical treatment of established PH and cor pulmonale have had limited success. The cornerstone of the treatment of cor pulmonale in the patient with a VAS is the prompt removal of the offending atrial catheter [10, 32, 63]. Lifelong anticoagulation therapy is currently recommended with the rationale to prevent in situ thrombosis and recurrent venous thromboembolism. Typically, vitamin K antagonists are preferred anticoagulants in CTEPH patients [49, 63]. Pulmonary thromboendarterectomy (PTA) is the gold standard of treatment in surgically eligible patients, and is the only treatment that offers a potential cure. The principle of PTA is to reduce the thromboembolic burden within the pulmonary vasculature as distal as possible in order to restore normal pulmonary blood supply and correct V/Q mismatch. Balloon pulmonary angioplasty is the only percutaneous interventional approach available as salvage therapy for patients with inoperable CTEPH [10, 49].

### Treatment of hydrocephalus

If atrial or pulmonary thromboembolus is found, it is recommended to remove the offending shunt [32]. However, the directly withdrawal of the distal catheter may lead to free the thrombus with risk of PE [14]. In general, the recommendations are to treat catheter-related thrombosis for 3 months with anticoagulation therapy followed by removal of catheter, or to directly remove the catheter by surgery [14]. Removal of the VAS may not be an option in all patients. There have been reports of the use of thrombolytic agents and/or anticoagulation therapy to correct malfunction caused by thrombosis [11, 14, 56]. But those patient are prone to have a recurrence of thrombosis following cessation of therapy [14]. After removal of the catheter, the shunting device should be replaced at an extravascular site, such as ventriculopleural shunts (VPLS), ventriculosinus shunts (VSS), or ventriculovesical shunts (VVS) [7, 9]. VPLS is a safe and viable second-line procedure for cases in which VPS are unsuitable. The current research results show that the complication rate of VPLS is comparable to that of VPS [64, 65]. The revision, infection, and survival rates between VAS and VPLS are similar, but it is free of the life-threatening cardiovascular complications [66]. However, VPLS may be not useful in infants owing to fear of complications such as tension hydrothorax and pneumothorax [67]. When the peritoneal and pleural cavity are impossible, the venous sinuses may be considered. Venous sinuses are the physiological drainage site. VSS have several advantages over the classic VPS and VAS. First, overdrainage is prevented by maintaining a natural, self-regulating antisiphon effect of the internal jugular vein [26]. Second, shunting the ventricles to a cranial sinus would avoid the flow problems since both are intracranial structures with minimal alteration of pressure gradient with posture and activity [68]. Thirdly, the shunt system is shorter in length and confined to the skull, which minimizes the risk of mechanical failure and infection [26]. Regarding immediate and early complications, no venous sinus thrombosis has been reported in patients with placement of the VSS, but follow-up period was too short in the majority of patients to make a valid comment on delayed complications with this type of shunts [68, 69]. Another possible option is VVS. Vesical shunting of CSF was first described in the management of hydrocephalus by Charles in 1980 [70]. Later studies have shown that VVS may be considered for the treatment of patients with hydrocephalus who are not candidates for a VPS or VAS [71, 72]. When the above shunt placement is not possible and a low CSF output is known, CSF diversion to the gall bladder may be an acceptable alternative [73].

## Prevention

VAS are at risk for different complications, and some of these are potentially life-threatening, but may be reversible with early and adequate diagnosis and treatment. Unfortunately, there are no systematical guidance recommendations on preventing these serious complications in patients after VAS placement. Recently, many attempts have been made to reduce the incidence of thromboembolic complications, including the improvement of shunt system, perfect preoperative preparation, accurate placement of catheter, and efficient follow-up.

### Shunt system

Several shunt-related factors might have an influence on clot formation, especially shunt material and design. The shunt material should be biocompatible and hemocompatible. Siliconized shunt tubing slightly decrease the incidence of thrombosis, but is not totally successful [12]. Different coatings could be used to further reduce the thrombogenicity of biomaterials. Typical examples are phosphorylcholine and heparin, both of which might be useful in reducing the general thrombogenicity of intravenous catheters and might also counteract the procoagulant effect of CSF [26]. Some researchers pioneered using retrograde insertion of distal catheters resulting in a constantly renewing CSF sleeve that prevents adherence of proteins and platelets to the foreign material’s surface [68, 69]. This theoretically reduces thrombus formation. Contradictory to the protective effect in theory, increased clot formation was observed on CSF-infused shunts in vitro, suggesting a CSF sleeve may be harmful [26]. Another problem is that there is a wake zone exist in the retrograde ventriculovenous shunts, characterized by a slow and nonlaminar flow, leading to more clot formation on the wake side of a shunt [26]. Therefore, avoiding placement of a shunt in a retrograde direction as far as possible can minimize contact between CSF and shunt material and prevent CSF from entering the distal shunt tip, reducing the risk of clot formation. If necessary, wake zones should be as small as possible by reducing the volume of the intravascular catheter. Ideally, when the distal shunt tip is oriented perpendicular to the blood flow, CSF will not drain along the shunt surface or into wake zones [26].

### Preoperative evaluation

Because of the relative high risk and severity of the thromboembolic complications in a VAS, numerous studies recommend to pursue a preoperative evaluation and thrombophilic screening in all patients being considered for VAS insertion [14, 32, 33]. Preoperative evaluation of the recipient vessels and heart is important as the presence of anatomical variation and physiologically structural defects are not uncommon [18, 67]. Routine screening of coagulation function and thromboelastography should be recommended in patients preparing for VAS. If possible, hereditary hypercoagulability workup also should be carried out, including tests of antithrombin III, protein C, and protein S activity; factor V Leiden, C677T MTHFR, and prothrombin G20210A mutations; plasma homocysteine level, lipoprotein A, antiphospholipid antibodies, anticardiolipin antibodies; and lupus anticoagulant testing [14]. It is important to note that a VAS may be unsuitable for those patients who are complicated by heart disease, respiratory disease, coagulation disturbance, hereditary hypercoagulability diseases, history of thrombosis, and scarring at the insertion site in the neck [74]. In addition, because of the high prevalence of PE and PH, indication of VAS has to be retained considering thromboembolic risk factors as SCV stenosis, current smoking, oral contraceptive, obesity, or intravenous alimentation [3, 21, 39].

### Placement of catheter

Safe and optimal catheter placement is important for those patients who are receiving VAS. Shunting functions optimally when the distal catheter is in the right atrium of the heart. The reasons may be as follows. Contractions of the right atrium may cause a siphoning effect to increase flow from the distal catheter. Moreover, the blood stream with rapid flow and constant turbulence around the distal catheter may prevent endothelialization and subsequent mechanical obstruction when the catheter is in the right atrium [1, 18]. Under such conditions, the hazard of clotting around the tubing is minimized [75]. A principle is to ensure that the tip of catheter is implanted into the vessel at an adequate length that it remains freely mobile, preventing stagnation of flow and thereby preventing thrombosis [32]. A long catheter may migrate into the inferior vena cava or tricuspid valve, causing pulmonary embolus. Likewise, a short catheter increases the risk of thrombosis of the SVC [12, 76]. Notably, as younger patients mature and grow into adulthood, the tip of the catheter may move out of the atrium and into the great vessels. Hence, monitoring the location of catheters will help to ensure it remains mobile distally, decreasing the risk of this complication [2, 32, 39]. Varieties of operational techniques have been developed to improve catheter placement, including bone landmarks, chest radiographs, electrocardiogram, and TEE [67, 74].

The location of the T-5 and T-6 vertebral body has been used as a marker for proper catheter placement in the right atrium [14, 67]. It is reported that patients with VAS in whom distal catheter tips located above T-4 were associated with a higher incidence of obstruction whereas atrial catheters located at or below T-7 were associated with development of bacteremia [67]. Therefore, the position of the distal catheter should be confirmed with the help of chest radiographs. The method most commonly used at present to assure correct placement utilizes radiopaque injection while roentgenograms are made in the operating room. This technique have clear limitations: the visualization of the tip of the catheter is highly dependent on its radiopacity, and the relationship between the osseous anatomical landmarks used to verify the position of the catheter may significantly vary among individuals [67]. The atrial catheter may be connected to an electrocardiograph. As the catheter is advanced, the P-wave increases progressively in amplitude until it reaches a peak of amplitude and then decreases again until it becomes negative when it moves away from the heart to the inferior vena cava. The catheter should be in the mid atrium when the P wave is biphasic [12, 74, 75]. The atrial catheter can also be connected to a pressure transducer and monitored as it is advanced. Once the pressure pattern changes from a right atrial to a right ventricular pattern, the catheter is pulled back until the atrial pattern is regained [12]. Although undoubtedly useful, both of these techniques require a correct interpretation of the recordings of the leads, which may be difficult sometimes. Furthermore, they do not provide real-time visualization of the advancement and final placement of the distal end of the catheter [67]. Therefore, echocardiography was introduced to identify the jugular vein patency, accurately localize the distal end of a VAS to the atrium, and assess the function and position of the VAS. Especially real-time TEE monitoring can be used less invasively, more accurately, quickly, and safely [67, 77, 78]. However, it should be noted that the risk of esophageal perforation is always present and demands extreme caution during advancement of the probe. This procedure should not be performed in patients with pharyngeal, esophageal disease, or esophageal varices [67].

### Postoperative management

**Fig. 2:**
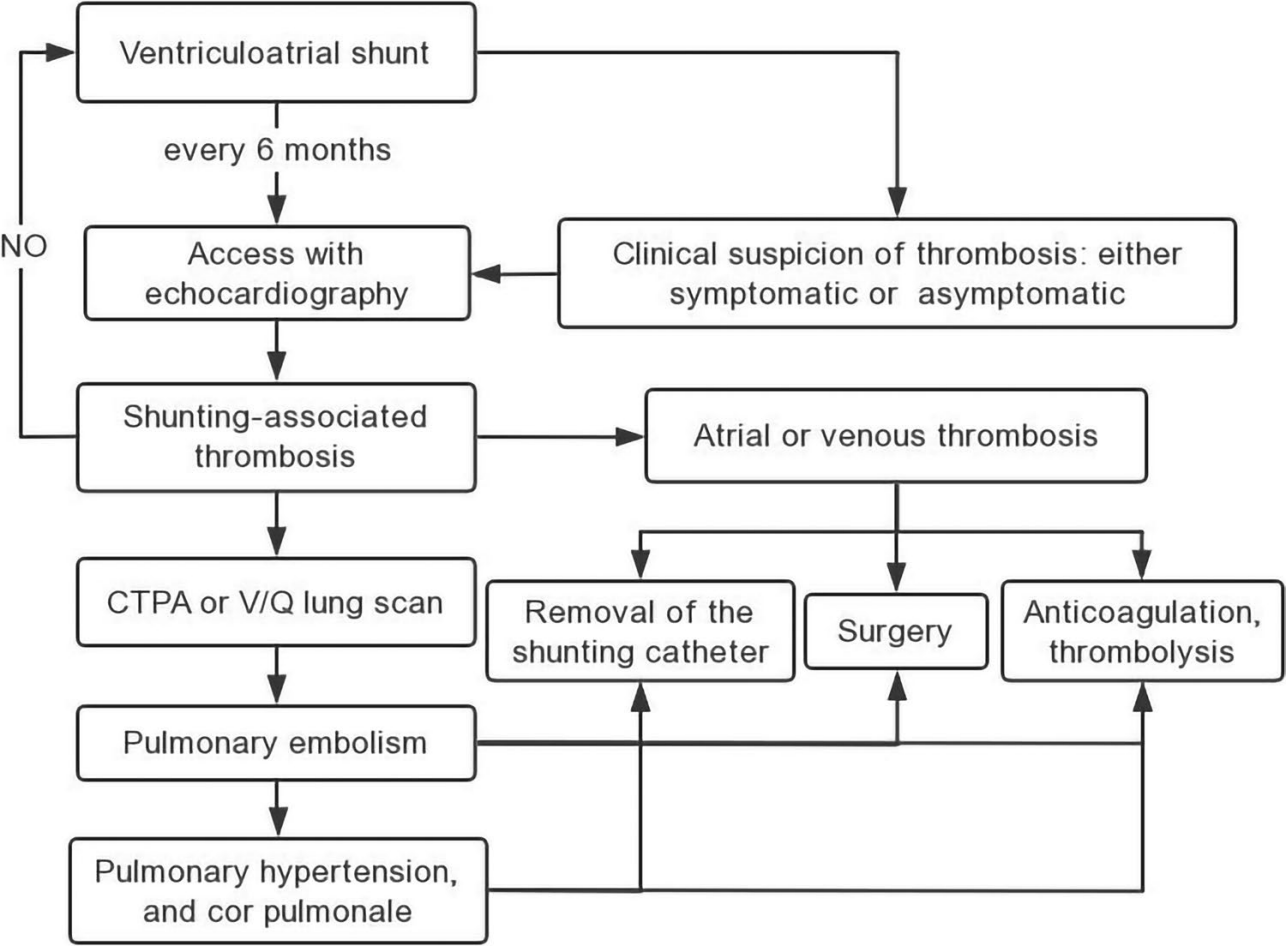
Follow-up strategy of ventriculoatrial shunt. CTPA, computer tomography pulmonary angiography; V/Q, ventilation-perfusion

A lifelong follow-up for preventing VAS-specific cardiopulmonary complications has been the present standard option (Fig. 2) [22]. A long period of asymptomatic intracavitary thrombi or PE usually precedes the terminal development of irreversible PH or cor pulmonale in VAS patients. Therefore, thromboembolic complications should be carefully monitored in all patients who have undergone VAS placement so that proper diagnosis and treatment of complications may be instituted early, thereby reducing morbidity and mortality [12]. Some publications have recommended to follow-up every patient who has undergone a VAS procedure twice a year [9, 10, 12]. At regular clinic follow-up appointments, thorough investigation of all pertinent history, cardiopulmonary symptoms, and physical examination in patients with VAS are imperative [12, 63]. Screening measures are logical as well. Clinical presentation is fundamental to help clinicians identifying suspected cases. Some patients may experience symptoms anytime from a few months to decades after placement of a VAS [4]. Cardiopulmonary symptoms must be elicited by deliberate questioning during the follow-up, and the physical findings associated with PH and right heart failure, such as a loud P2, a right-sided presystolic gallop, jugular venous distention, lower limb edema, and hepatomegaly must be sought warily. Most notably, even asymptomatic patients may have signs of cardiac enlargement [79]. When thromboembolic complications are suspected in a VAS patient, tests should include blood count, coagulation function, and radiologic studies [32]. Routinely, periodic chest radiographs for catheter position and pulmonary changes must be reviewed every 6 months after placement of a shunt [63]. An electrocardiogram is usually performed to rule out congestive failure. Importantly, an echocardiography may be essential screening technique to periodic clinical assessment, as it has the potential to assess the location of the tip of catheter and detect intracardiac thrombi and the late structural changes of the heart and pulmonary vasculature associated with occult PH [63]. When the catheter tip rises to the level of the upper part of the right atrium or in the lower superior vena cava, elective prophylactic lengthening of the atrial catheter is recommended [1]. If any suggestion of thromboemboli arises, a CT pulmonary angiography scan should be obtained [12]. Meanwhile, the shunt should be immediately removed, and patients should be treated medically or surgically depending on their medical condition and the characteristics of the thrombus. An extravascular site for the repositioning of the shunting device is recommended and close patient follow-up is needed [9, 10, 12].

### Prophylactic therapy

The higher incidence of thromboembolic complications in VAS patients prompted researchers to meditate on the significance of prophylactic therapy. Anticoagulation is widely used in patients with extraneous material in the blood system to avoid thromboembolic complications. Mostly, the anticoagulation could pause the epithelialization course of those materials and reduce the thromboembolic risk [18]. Literature has a mention of the use of prophylactic anticoagulants for VAS patients, and the authors found that the incidence of distal blockage was nearly three times higher in controls than in those who had undergone anticoagulant therapy, suggesting that anticoagulant therapy seems to have a real effect on susceptibility to shunt-related thrombosis [52]. Recently, a study of 6 patients also showed that the use of low-dose aspirin in VAS is safe and promising because none of the shunts had malfunctioned and any cardiac issues reported during follow-up [22]. Prior to that, the therapeutic use of acetylsalicylic acid and dipyridamole has been investigated, but the results were discouraging [10]. However, data on the efficacy and safety of prophylactic therapy in patients after VAS placement is lacking so that whether anticoagulation therapy should be continued in the presence of an indwelling VAS is currently unknown. Larger studies are needed to prospectively investigate a possible role for prophylactic anticoagulation or antiplatelet therapy.

## Conclusion

In despite of recent advances in the recognition of VAS-related thrombosis, diagnosis and management are still difficult because of the absence of early perceptible signs and a uniform treatment approach. The subtle cardiopulmonary presentation in a VAS patient deserves prompt attention since identification of such features will benefit subsequent diagnosis and intervention. However, the diagnostic work-up of shunting-related thrombosis usually bases on clinical suspicion and imaging confirmation starting with echocardiography. Because of relatively asymptomatic thromboembolism and increased risk of irreversible PH, prevention seems to be more important than treatment. Therefore, regular and specific follow-up examinations are strongly recommended in all VAS patients so that proper diagnosis and management of complications may be initiated early, thereby reducing morbidity and mortality. With the development of surgical technique and shunt design, these drawbacks may be overcome in the near future.

## Data Availability

Not applicable.
